# Novel Airfoil-Shaped Radar-Absorbing Inlet Grilles on Aircraft Incorporating Metasurfaces: Multidisciplinary Design and Optimization Using EHVI–Bayesian Method

**DOI:** 10.3390/s25144525

**Published:** 2025-07-21

**Authors:** Xufei Wang, Yongqiang Shi, Qingzhen Yang, Huimin Xiang, Saile Zhang

**Affiliations:** School of Power and Energy, Northwestern Polytechnical University, Xi’an 710129, China; xufei_wang@mail.nwpu.edu.cn (X.W.); qzyang@nwpu.edu.cn (Q.Y.); hmxiang@mail.nwpu.edu.cn (H.X.); zsle@mail.nwpu.edu.cn (S.Z.)

**Keywords:** aircraft engine inlet, radar-absorbing inlet grille (RIG), metasurface, NACA airfoil, Bayesian optimization

## Abstract

Aircraft, as electromagnetically complex targets, have radar cross-sections (RCSs) that are influenced by various factors, with the inlet duct being a critical component that often serves as a primary source of electromagnetic scattering, significantly impacting the scattering characteristics. In light of the conflict between aerodynamic performance and electromagnetic characteristics in the design of aircraft engine inlet grilles, this paper proposes a metasurface radar-absorbing inlet grille (RIG) solution based on a NACA symmetric airfoil. The RIG adopts a sandwich structure consisting of a polyethylene terephthalate (PET) dielectric substrate, a copper zigzag metal strip array, and an indium tin oxide (ITO) resistive film. By leveraging the principles of surface plasmon polaritons, electromagnetic wave absorption can be achieved. To enhance the design efficiency, a multi-objective Bayesian optimization framework driven by the expected hypervolume improvement (EHVI) is constructed. The results show that, compared with a conventional rectangular cross-section grille, an airfoil-shaped grille under the same constraints will reduce both aerodynamic losses and the absorption bandwidth. After 100-step EHVI–Bayesian optimization, the optimized balanced model attains a 57.79% reduction in aerodynamic loss relative to the rectangular-shaped grille, while its absorption bandwidth increases by 111.99%. The RCS exhibits a reduction of over 8.77 dBsm in the high-frequency band. These results confirm that the proposed optimization design process can effectively balance the conflict between aerodynamic performance and stealth performance for RIGs, reducing the signal strength of aircraft engine inlets.

## 1. Introduction

To improve aircraft survivability, stealth technology, aimed at reducing the radar cross-section (RCS), has been widely applied to aircraft shape and structural design [1]. Since the engine inlet, containing complex structures such as compressor fans, becomes the primary forward scattering source for aircraft [2], the stealth design of the inlet has emerged as a key technology in advanced aircraft development. Nevertheless, inlet design must consider not only stealth performance but also aerodynamic characteristics, making forward stealth design a complicated engineering problem.

Three approaches are commonly used to reduce the electromagnetic wave echo intensity of engine inlets. The first approach involves applying coating materials on intake cavity walls to absorb electromagnetic waves [3,4,5,6]. The second approach employs an serpentine inlet duct with a large offset so that electromagnetic waves cannot directly impinge on highly reflective end surfaces [7,8,9,10]. The third approach uses a metallic grille or other blocking measures at the inlet to shield it from incident and reflected electromagnetic waves [11,12,13,14,15,16,17]. Conventional coating materials often lead to a significant increase in weight [18], whereas the large-offset serpentine inlets require more geometric space in the offset direction. Consequently, grille-based shielding is a more convenient stealth enhancement for the original design.

Both the F-117A and RQ-170 low-observable aircraft adopt metallic grilles to shield the inlet cavity; however, these grilles only exhibit satisfactory screening effects at specific frequencies [2], failing to address the full-spectrum stealth requirements of modern battlefields. Current research on new electromagnetic metamaterials indicates that such materials display various complex effects and phenomena rarely found in natural materials [19,20], such as a negative refractive index [21,22,23], polarization conversion [24,25], perfect absorption [26,27], and electromagnetic invisibility [28,29,30]. Radar-absorbing structures (RAS) based on spoof surface plasmon polaritons (SSPP) have recently become a focal point of research. These novel absorbing structures achieve the controlled excitation and propagation of surface plasmons by creating particular patterns on metal or dielectric substrates [31]. Thus, electromagnetic metasurfaces exhibit tremendous potential for application in improving engine inlet grilles.

In the field of RAS research based on metamaterials, Sheokand [32] introduced an optically transparent, broadband microwave absorber fabricated from ITO resistive thin-film etching units, achieving over 10 dB of absorption within the 8.6–12.4 GHz bandwidth. Subsequently, Sheokand [33] adopted a flexible substrate to modify the resistor film structure, enabling over 90% absorption from 4.0 GHz to 17.20 GHz. Wang [25] proposed a sandwich structure where a polarization–conversion metasurface and a metamaterial absorber reduced the RCS by more than 10 dB over the 2–35 GHz range. Yu [34] presented an SSPP-based absorber composed of inward-bending metal strips with lumped resistors, achieving over 80% broadband absorption under normal incidence. Shen [35,36,37] proposed horizontal, vertical, and three-dimensional zigzag strip designs, leveraging bent metallic lines to enhance SSPP dispersion tuning. This enables strengthened lossy absorption in finite dimensions and improves low-frequency absorption. Zhou [38] described a hybrid absorber composed of SSPP structures and planar resistive metasurfaces, offering wide and stable absorption under broad incidence angles and achieving 90% absorption within a 6.7 GHz bandwidth. Yang [39] introduced a broadband microwave absorber with optical transparency, heat insulation, and soundproofing. Leveraging ITO-based metamaterials, this device achieves over 90% microwave absorption from 5.6 GHz to 23 GHz (covering both the X and Ku bands). Yang [40] further designed a transparent, broadband microwave metasurface absorber based on a silver nanowire resistor thin film that attains above 90% absorption from 4.1 to 18.2 GHz with 75% visible light transparency. Hence, employing metasurface principles offers a pathway to improve engine inlet grilles.

As for evolutionary algorithms, although modern evolutionary algorithms are popular for multi-objective optimization with no gradient information [41,42,43], they require numerous and expensive function evaluations. By contrast, Bayesian optimization integrates prior beliefs about the problem to guide the sampling of new data while balancing exploration and exploitation, making it a leading technique to tackle costly black-box problems [44,45]. Laumanns [46] compared prevalent evolutionary algorithms with Bayesian optimization, demonstrating that Bayesian optimization can find Pareto solutions in fewer trial instances and often dominates other algorithms in terms of solution sets. Saves [47] introduced a hyperparameter-adaptive process to improve Bayesian optimization, achieving marked improvements over genetic algorithms. Zhang [48] proposed a multi-objective Bayesian optimization framework for hybrid design optimization to enhance the aerodynamic and infrared characteristics of a serpentine nozzle, confirming that this method effectively addresses multi-objective problems in aircraft design. Therefore, Bayesian optimization methods appear suitable for the multidisciplinary aerodynamic and electromagnetic optimization of grilles.

Although metasurface-based absorbers exhibit excellent radar-absorbing capabilities, current research mainly focuses on designs with a metal back-reflector or dielectric absorber backing configurations that do not allow airflow. The application of metasurface principles to RIGs remains scarce. Furthermore, absorber research has largely centered on rectangular cross-section substrates, whereas such cross-sections degrade the aerodynamic performance for inlet grilles. A metasurface radar-absorbing inlet grille based on a NACA symmetric airfoil could potentially absorb electromagnetic waves while mitigating aerodynamic performance losses. To the best of our knowledge, no significant research has yet been conducted on the combination of RIGs with the multi-objective optimization of both aerodynamic and electromagnetic performance.

To address the high aerodynamic losses caused by incorporating conventional absorbers into engine inlet grilles, this paper proposes a design solution that simultaneously ensures broadband absorption and low flow loss. By integrating NACA symmetric airfoils with metasurface design, a novel airfoil-shaped RIG is acquired. Subsequently, a multi-objective optimization framework is proposed to address the aerodynamic and electromagnetic properties of the airfoil-shaped RIG, providing a set of non-dominated optimal solutions. Then, representative optimal solutions are compared with baseline models and improved models to assess the performance differences. Finally, a coupled simulation loads the proposed design into an engine inlet to validate the effectiveness of the airfoil-shaped RIG. Specifically, Section 2 discusses the research methods and formulas for aerodynamic and electromagnetic simulations. Section 3 introduces the theory of Bayesian optimization and its convergence validation. Section 4.1 defines the problem and the framework of this optimization. Section 4.2 presents multidisciplinary optimized results for the baseline and the proposed airfoil-shaped RIGs. Section 4.3 illustrates the performance of the optimized RIG integrated into the aircraft inlet. Section 5 concludes this article.

## 2. Design Theory and Numerical Methods

### 2.1. Design Theory of RIGs

Surface plasmon polaritons (SPPs), as a unique electromagnetic propagation mode, have been primarily studied in the optical frequency range. In these optical regimes, certain metals (e.g., gold and silver) have negative permittivity, supporting SPP excitation on metal–dielectric interfaces. Meanwhile, when transitioning to microwave frequencies, most metals behave as near-perfect electric conductor (PECs), so their surfaces no longer support conventional SPP modes.

Researchers have addressed this limitation by introducing spoof surface plasmon polaritons (SSPPs), an artificially engineered structure intended to mimic natural SPPs in the microwave frequency range. SSPPs are typically achieved by etching subwavelength patterns on metallic surfaces. This artificial structure supports a specific type of surface wave, which exhibits confinement and rapid attenuation perpendicular to the interface, with its field distribution on both sides of the interface demonstrating evanescent decay. However, SSPP operation is restricted to the transverse magnetic (TM) mode, as only TM waves possess the electric field component perpendicular to the interface required to induce surface charge polarization. Transverse electric (TE) waves cannot support SSPP propagation due to the absence of such a perpendicular electric field component. Given that a metasurface absorber employing SSPPs might be subjected to various polarization modes, an appropriate array design is necessary to ensure that the grille can maintain TM propagation under different polarizations.

Based on waveguide transmission line theory, the attenuation constant $\alpha_{T}$ of spoof surface plasmons may be expressed as [49]

(1) $$\alpha_{T}=\sqrt{k_{x}^{2}-k_{0}^{2}}$$

where $k_{x}$ is the wavenumber in the propagation direction (the *x*-axis), and $k_{0}$ is the wavenumber in air. Because SSPPs confine electromagnetic energy near the interface for propagation, the transverse electromagnetic field energy exhibits exponential decay. With $k_{0}$ unchanged in air, $\alpha_{T}$ grows as $k_{x}$ increases, meaning that a larger $\alpha_{T}$ value denotes a stronger capacity for confining the electromagnetic field. Consequently, the proposed metasurface grille is a slow-wave structure.

### 2.2. Model Description

To achieve wideband radar microwave absorption for engine inlet applications, we base the model design on a periodically arranged comb-shaped metal strip [34] for spoof surface plasmon polaritons, sandwiching an ITO resistive film in the middle layer to broaden the absorption bandwidth.

Although a conventional rectangular cross-section radar-absorbing grille presents strong electromagnetic absorption, it will introduce large amounts of flow loss that reduce the engine intake efficiency and thrust performance of the full aircraft engine. This means that the aerodynamic losses caused by the grille conflict with its electromagnetic stealth capabilities. In order to solve this issue, utilizing metasurface theory, we propose a novel airfoil-shaped radar-absorbing grille by incorporating the NACA family of symmetric airfoil profiles, aiming to maintain favorable absorption while reducing the flow loss that is caused by rectangular-shaped grilles.

**Figure 1:**
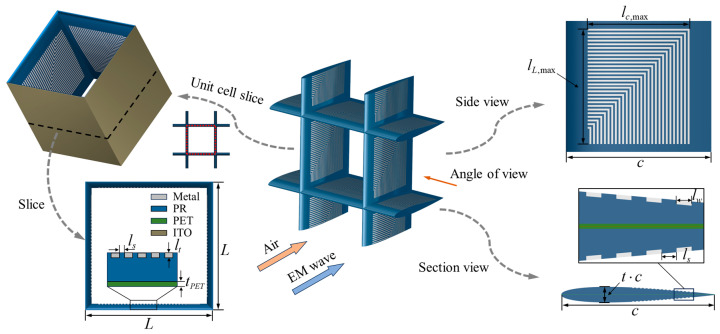
Schematic diagram of three-dimensional and sliced RAS model.

Figure 1 shows the three-dimensional configuration of the proposed airfoil-shaped RIG, where the rectangular grille differs in its cross-sectional shape. The airfoil-shaped RIG comprises a symmetric airfoil dielectric substrate and a continuously varying zigzag metal strip array, with an embedded resistive film. Specifically, the dielectric substrate forms the main structure of the absorber. The zigzag metal strip array is projected onto the substrate at predetermined dimensions, and the resistive film is sandwiched between the substrate layers. PET is used for the substrate, with relative permittivity $eps_{pet}=3.2$ and loss tangent tanΔ=0.06. The copper strip array is treated as a perfect electric conductor (PEC). The ITO material is sputtered onto the PET film, thus forming an ITO resistive film. Table 1 summarizes the main design parameters and their notations; a typical set of values is selected to define the baseline model of the airfoil-shaped RIG.

**Figure 2:**
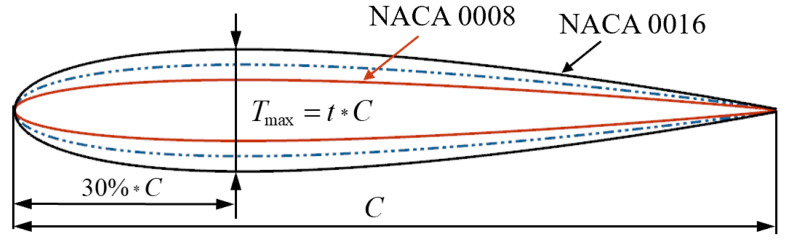
NACA airfoil profile of proposed model.

As depicted in Figure 2, the design references NACA0008 through NACA0016, whose maximum thickness is located at the 30% chord position, ranging continuously from 8 to 16% of the chord length. Based on the variation rules of the series of airfoil profiles, the airfoil thickness can be adjusted continuously within the range of real numbers.

### 2.3. Aerodynamic Computation

In this study, three-dimensional compressible steady-state RANS equations are solved in ANSYS Fluent 19.2 to investigate the gas flow around the RIG. A second-order upwind scheme is adopted for discretization, and a density-based solver is used. The no-slip boundary condition is imposed on surfaces, and the k-ω SST turbulence model is selected to capture relevant flow features more accurately. To enable automated simulation and optimization, an unstructured mesh is generated for the computational domain, with mesh refinement applied to the grille wake zone. To ensure an accurately resolved boundary layer, 12 boundary layer grids are used, with the first layer set to 0.004 mm in thickness to satisfy y+ around 1 with a growth ratio of 1.2. The total mesh size is around 9.5 million cells.

**Figure 3:**
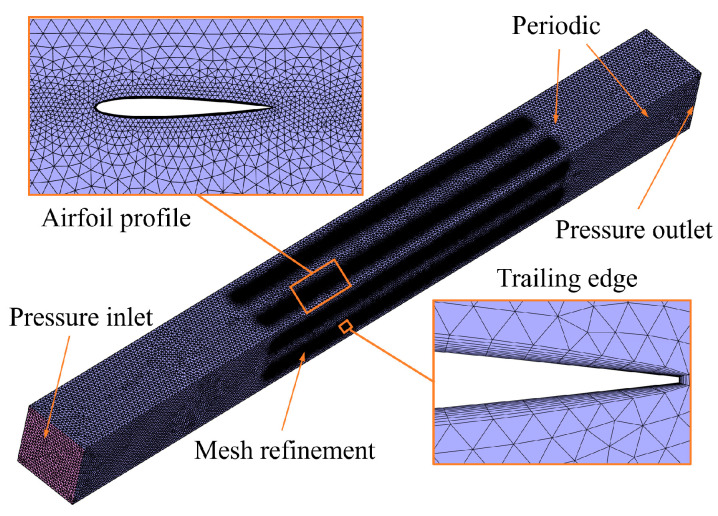
Aerodynamic calculation region and detailed meshing schematic.

As depicted in Figure 3, a “#” hash-shaped periodic unit is used to represent the flow domain. This model is suitable for capturing complete flow characteristics under an equivalent assumption. Periodic boundary conditions are applied on the four surrounding sides to simulate an infinitely large grille. The domain extends 10*c* upstream and 15*c* downstream of the grille’s leading and trailing edges, respectively, where *c* is the chord length of a single grille unit. To compare the aerodynamic characteristics of different models under 11-km-altitude flight conditions, the specific boundary conditions shown in Table 2 are considered.

To validate the accuracy of the turbulence model, comparative verification was conducted using the experimental results of the modified NACA 653-019 airfoil from the Langley Memorial Aeronautical Laboratory in the United States. Compared to the original airfoil, the modified version features a linear correction at 0.2*c* of the trailing edge. In this experiment, a smaller airfoil (127 mm) was used compared to a full-scale wing, and the test involved a relatively high Mach number (0.442 Ma), being similar to the operating conditions of the airfoil studied in this paper. Therefore, this serves as an appropriate validation case.

**Figure 4:**
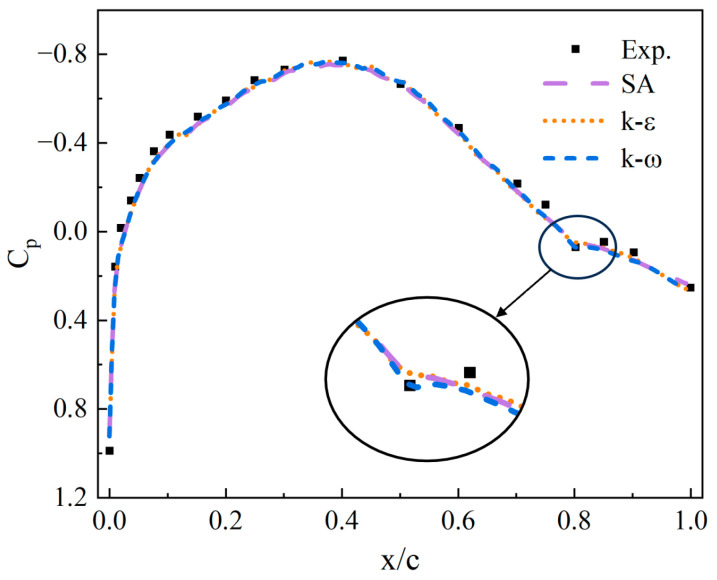
Comparison between different turbulence models.

The comparison of the surface pressure distribution curves in Figure 4 between the experiment and simulation shows that the simulation results obtained using the k−ω SST turbulence model closely match the experimental data. The calculated pressure coefficients deviate by less than 3.39% from the experimental data across most of the airfoil surface. This demonstrates the capability of the model to accurately capture the overall trend of the surface static pressure distribution, proving the applicability of the turbulence model to similar flow problems.

### 2.4. Electromagnetic Computation

Electromagnetic wave propagation in dielectrics is governed by the following Maxwell’s equations:

(2) $$\left\{\begin{array}{c} \nabla \times \vec{H}=\vec{J}+\frac{\partial \vec{D}}{\partial t}\\ \nabla \times \vec{E}=-\frac{\partial \vec{B}}{\partial t}\\ \nabla \cdot \vec{B}=0\\ \nabla \cdot \vec{D}=\rho \end{array}\right.$$

where $\vec{E}$ and $\vec{H}$ are the electric and magnetic fields; $\vec{D}$ and $\vec{B}$ are the electric displacement and magnetic induction, respectively; and $\vec{J}$ is the current density.

In this study, the finite element method (FEM) in CST Studio 2022 is used for the electromagnetic simulation of the RIG. Owing to the grille’s periodic configuration, Floquet theory is needed to solve Maxwell’s equations. According to Floquet’s theorem, wave propagation in a 2D periodic structure can be expressed as the product of a periodic function and an exponential function.

**Figure 5:**
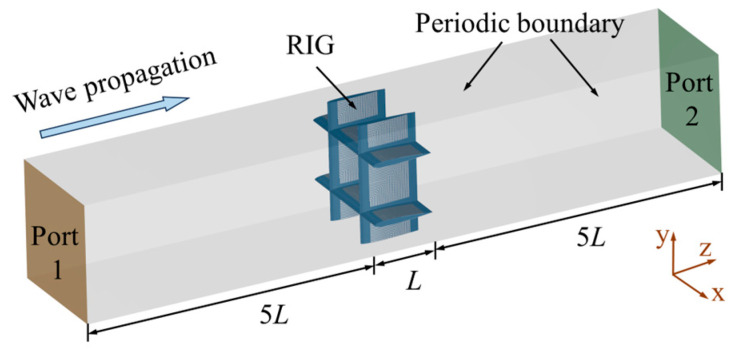
Schematic of electromagnetic calculation and boundary conditions.

This study uses a periodic unit structure as the computational model to simulate the electromagnetic characteristics of the proposed RIG. Figure 5 illustrates the detailed setup of the computational model. Periodic boundary conditions are applied in the x and y directions to simulate an infinite inlet grille. The z direction is defined as open space to simulate the free propagation of electromagnetic waves in a real environment. Excitation ports 1 and 2 are set on the two end faces in the z direction. The distance from the absorbing structure to the excitation/reception ports in the z direction is 5 times the unit spacing *L*. The calculation frequency range is 2–18 GHz, covering the key detection frequency range for aircraft applications.

### 2.5. Performance Metrics and Formulas

In terms of aerodynamics, to describe the flow capacity of the RIG, the ratio of the work energy of the fluid after passing through the grille to that before entering the grille is used as a measure, and the total pressure recovery coefficient is defined as [50]

(3) $$\sigma =P_{t,out}/P_{t,in}$$

where $P_{t,out}$ and $P_{t,in}$ are the total pressures at the outlet and inlet, respectively.

In terms of electromagnetics, to describe the absorption performance of the RIG, the absorption rate α(f) as a function of the electromagnetic wave frequency f is defined based on the law of energy conservation according to Refs. [51,52]:

(4) $$\alpha (f)=1-|S_{11}(f){|}^{2}-|S_{21}(f){|}^{2}$$

where $S_{11}(f)$ is the frequency-dependent reflection coefficient and $S_{21}(f)$ is the frequency-dependent transmission coefficient.

Since the radar-absorbing grille has a transmission relationship, the complete material impedance characteristics as a function of the frequency are calculated as [53]

(5) $$Z=\frac{(T_{22}-T_{11})+\sqrt{{\left(T_{22}-T_{11}\right)}^{2}+4T_{12}T_{21}}}{2T_{21}}$$

where *T*-matrix elements can be expressed by

(6) $$\left\{\begin{array}{l} T_{11}=\frac{(1+S_{11})(1-S_{22})+S_{21}S_{12}}{2S_{21}}\\ T_{12}=\frac{(1+S_{11})(1+S_{22})-S_{21}S_{12}}{2S_{21}}\\ T_{21}=\frac{(1-S_{11})(1+S_{22})-S_{21}S_{12}}{2S_{21}}\\ T_{22}=\frac{(1-S_{11})(1+S_{22})+S_{21}S_{12}}{2S_{21}} \end{array}\right.$$

To quantitatively describe the broadband absorption performance of the radar-absorbing grille under different design parameters, the maximum continuous 90% absorption bandwidth within the calculated frequency range is characterized as

(7) $$\begin{array}{l} \Delta f_{c,90\%}=\max\{f_{2}-f_{1}:f_{1},f_{2}\in [f_{\min},f_{\max}],\\ \;\;\;\;\;\;\;\;\;\;\;\;\;\;\;\;\;\alpha (f)>0.9\;\mathrm{for}\;\mathrm{all}\;f\in [f_{1},f_{2}]\} \end{array}$$

where the frequency range is from $f_{\min}$ to $f_{\max}$, which is set as 2–18 GHz in this paper. There is potential to exceed 90% of the absorption bandwidth, and the symbol $\Delta f_{c,90\%}$ denotes the maximum bandwidth. Then, the relative proportion of the continuous 90% absorption bandwidth is

(8) $$\eta_{\Delta f_{c,90\%}}=\Delta f_{c,90\%}/\Delta f_{max}$$

where $\Delta f_{\max}$ is the maximum possible bandwidth in the range of interest.

## 3. Bayesian Optimization

### 3.1. Optimization Theory

Bayesian optimization is a global optimization approach for expensive black-box functions. It builds a probabilistic model of the objective function and uses an acquisition function to balance the exploration of the search space and the exploitation of identified promising regions. The main problem that it tackles is

(9) $$x^{*}=\underset{x\in X}{\arg\max}f(x)$$

where $x^{*}$ is the set of optimal solutions, and X is the design space defined in search space ${\mathbb{R}}^{d}$. $f:X\to {\mathbb{R}}^{m}$ is the black-box function set to be optimized. The objective function f(x) can be written as a set $\left\{f_{1}(x),f_{2}(x),\ldots ,f_{m}(x)\right\}$.

This section demonstrates how to marginalize weight vectors in Bayesian linear regression and apply kernel trick-based Bayesian nonparametric regression. Suppose that the observation noise variance σ^2^ is fixed, and the regression coefficients w have a zero-mean Gaussian prior $p(w|v_{0})=N(0,v_{0})$. To keep the Gaussian property, integrating out w analytically yields

(10) $$\begin{array}{c} p(Y|X,\sigma^{2})=\int p(Y,w|X,\sigma^{2})dw\\ \;\;\;\;\;\;\;\;\;\;\;\;\;\;\;\;\;\;\;\;\;\;\;\;\;\;\;\;\;\;\;\;\;\;\;\;=\int p(Y|X,w,\sigma^{2})p(w|X,\sigma^{2})dw\\ \;\;\;\;\;\;\;\;\;\;\;\;\;\;\;\;\;\;\;\;\;\;\;=N(Y|0,Xv_{0}X^{T}+\sigma^{2}I). \end{array}$$

where X is the design matrix, Y is the observation vector, and I is the identity matrix.

The famous kernel trick uses a kernel function k to compute these inner products. This approach not only allows one to calculate the marginal likelihood of the training data but also enables posterior predictions $y^{*}$ of any new input $x^{*}$:

(11) $$p(y^{*}|x^{*},X,Y,\sigma^{2})=\frac{p(y^{*},Y|x^{*},X,\sigma^{2})}{p(Y|X,\sigma^{2})}$$

### 3.2. Gaussian Process

By kernelizing the marginalized Bayesian linear regression, a Gaussian process (GP) emerges. A $GP(\mu_{0},k)$ is fully specified by its prior mean function $\mu_{0}:X\to R$ and its positive-definite kernel or covariance function k:X×X→R. Consider a finite set of n design points $x_{1:n}$ and let $f_{i}:=f(x_{i})$. Under GP regression, we assume that $f:=f_{1:n}$ has a joint Gaussian distribution, and, given f, the observations $y:=y_{1:n}$ are also normal:

(12) f | X~N(m,K)

where the mean vector and covariance matrix elements are $m_{i}:=\mu_{0}(x_{i})$ and $K_{i,j}:=k(x_{i},x_{j})$. As for multi-objective Bayesian optimization, each function is constructed by independent Gaussian process priors,

(13) $$f_{i}(x)\sim N(m_{i}(x),k_{i}(x,x^{\prime }))$$

In GP regression, the covariance function k controls the smoothness of the modeled response function. The Matérn family of kernels is parameterized by a smoothness parameter ν. Samples from a GP with a Matérn kernel are ν-times differentiable. In this paper, we employ the Matérn kernel

(14) $$k(x,x^{\prime })=\theta_{0}^{2}\exp(-\sqrt{5}r)(1+\sqrt{5}r+\frac{5}{3}r^{2})$$

where $r^{2}={\left(x-x^{\prime }\right)}^{T}\Lambda (x-x^{\prime })$, and Λ is a diagonal matrix of length scales. The amplitude and length scales of Λ collectively form the hyperparameters θ.

### 3.3. Acquisition Function

In Bayesian optimization, the acquisition function is used to balance the exploration of the search space and the exploitation of the current best region. After each iteration of Bayesian optimization, the next sampling point in the design space is determined by maximizing the acquisition function. For a finite approximate Pareto front P, the hypervolume (HV) is the Lebesgue measure of the space between P and a known reference point r. When the Pareto front is 2D, we have

(15) $$HV(P|r)=\lambda_{M}(U_{v\in P}[r,v])$$

where [r,v] denotes the hyperrectangle bounded by the vertices r and v. The hypervolume improvement (HVI) of a set of observation points Y relative to the existing approximate Pareto front P and reference point r is defined as

(16) HVI(Y|P,r)=HV(P∪Y|r)−HV(P|r)

Calculating HV requires computing the volume of a typically non-rectangular polyhedron, and its time complexity grows super-polynomially with the number of objectives. Since the function values of unobserved points are unknown in black-box optimization, the HVI of out-of-sample points is also unknown. This process requires integration in high-dimensional space, making direct computation difficult. Monte Carlo methods are typically used for approximation:

(17) $$EHVI(Y|P,r)=\frac{1}{N}\sum_{j=1}^{N}\left[HV(P\cup y_{i}|r)-HV(P|r)\right]$$

where $y_{i}$ is a sample from the posterior distribution at position x.

### 3.4. Optimal Convergence Verification

To verify the convergence of EHVI-based Bayesian optimization, we use the Branin-Currin and ZDT2 problems as test optimization problems. The Branin-Currin problem has two design variables and two objective functions, while the ZDT2 problem has four design variables and two objective functions, similar to the multi-objective optimization problem studied in this paper. Sixteen initial sample points are obtained using the Sobol method. Since heuristic algorithms such as NSGA2 and MOEA/D require a large number of sample points in every iteration and thus expensive function calls, they are not suitable for the scope of this study. Random search, ParEGO, and EHVI are used as acquisition functions for iterations of Bayesian optimization. The logarithmic hypervolume difference is used as the evaluation metric, expressed as

(18) $$\log_{10}\Delta HV=\log_{10}(HV_{true}-HV_{current})$$

where $HV_{true}$ is the true Pareto front’s hypervolume and $HV_{current}$ is the hypervolume found at the current iteration. Thus, $\log_{10}(HV_{true}-HV_{current})$ measures convergence to the true front.

**Figure 6:**
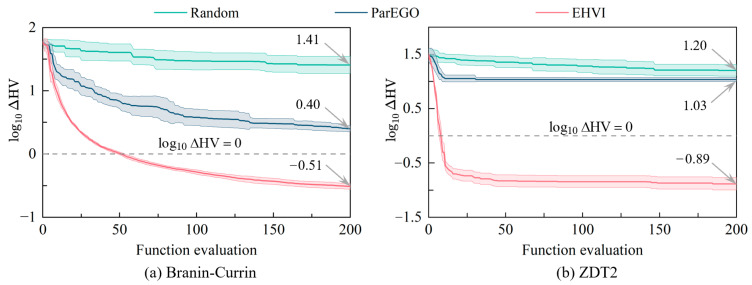
Hypervolume convergence of standard test functions.

Figure 6 plots the mean hypervolume differences for Branin-Currin and ZDT2 after 200 iterations. After initialization, all three methods show decreasing hypervolume differences with increasing iterations. For Branin-Currin, EHVI, ParEGO, and random search achieve −0.51, 0.40, and 1.41 at iteration 200, respectively. For ZDT2, the results are −0.89, 1.03, and 1.20, respectively. EHVI’s Pareto frontier is closest to the true solution, outperforming ParEGO and random search. Hence, we use EHVI-based BO for multi-objective optimization in this study.

Moreover, Figure 6 shows the average hypervolume difference $\log_{10}\Delta HV$ as a function of the number of iterations for the Branin-Currin and ZDT2 problems after 10 runs of the different methods. It can be seen that, after the initialization of the sample points, $\log_{10}\Delta HV$ decreases with the number of iterations for all three methods. For the Branin-Currin problem, the $\log_{10}\Delta HV$ values for EHVI, ParEGO, and random search after 200 iterations are −0.51, 0.40, and 1.41, respectively. For the ZDT2 problem, the $\log_{10}\Delta HV$ values for EHVI, ParEGO, and random search after 200 iterations are −0.89, 1.03, and 1.20, respectively. This indicates that the Pareto boundary obtained by the EHVI acquisition function is closer to the true solution set, significantly outperforming the ParEGO and random strategies. Therefore, this paper uses EHVI-based Bayesian optimization as the research method.

## 4. Results and Discussion

### 4.1. Optimization Design

To simultaneously achieve better absorption performance and an excellent flow capacity, this section uses the $Matern_{5/2}$ kernel function in the Gaussian process as the surrogate model and employs the multi-objective Bayesian optimization algorithm based on the EHVI acquisition function to optimize the design parameters of the airfoil-shaped RIG, aiming to find a design with superior electromagnetic and aerodynamic performance.

In the optimization, the NACA0008 to NACA0016 airfoils are used as the basic airfoil shapes, with a maximum thickness of 8–16% of the chord length *c*, a chord length range of 8–16 mm, a grille unit spacing range of 8–16 mm, and a resistive film resistance range of 1−800 Ω/□, where the unit Ω/□ means omega per square. The optimization problem can be described as

(19) $$\begin{array}{c} \max:f(L,C,T,\rho )=\{\sigma ,\eta_{\Delta f_{c,90\%}}\}\\ s.t.:\;L\in [8,16]\;\mathrm{mm}\\ \;\;\;\;\;\;\;\;\;C\in [8,16]\;\mathrm{mm}\\ \;\;\;\;\;t\;\in [8,16]\;\%\\ \;\;\;\;\;\;\;\;\;\;\;\;\rho \in [1,800]\;\Omega /□ \end{array}$$

where σ and $\eta_{\Delta f_{c,90\%}}$ are defined in Equations (3) and (8), respectively. For airflow through any non-working structure, the total pressure recovery coefficient σ∈[0,1] is limited. The electromagnetic wave frequency calculation interval is 2–18 GHz, so $\Delta f_{c,90\%}\in [0,16]$ and $\Delta f_{\max}=16\;\mathrm{GHz}$ are defined accordingly.

**Figure 7:**
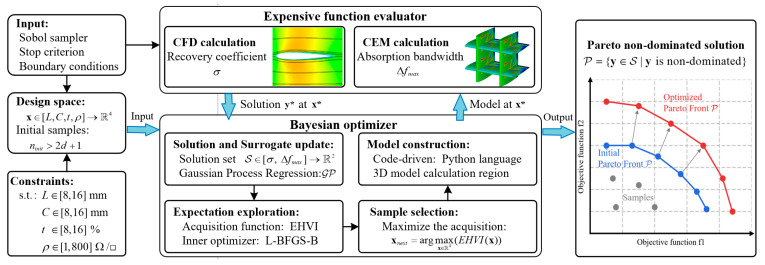
Flowchart of aerodynamic–electromagnetic multi-objective Bayesian optimization.

Figure 7 shows the EHVI-driven aerodynamics and electromagnetics multi-objective optimization workflow. In this optimization design study, the Sobol method is first used to sample 16 initial samples from the design space using a low-discrepancy sequence. Three-dimensional modeling software is used to design the geometric models based on the sample parameters. The initial sample points’ aerodynamic and electromagnetic characteristics are then evaluated using CFD and CEM methods. A Gaussian process is used to construct the surrogate model based on the observed data, and the initial sample data solution set is established. The EHVI acquisition function is used to obtain the next set of observation points with the maximum expected hypervolume improvement probability, and the modeling software and simulation tools are used to conduct the calculation study. The latest observation values are then substituted into the surrogate model, and the sample data solution set is updated. This process is iterated until the termination conditions are met, and the Pareto non-dominated solution set is output. This paper uses EHVI to collect 100 additional sample points and employs the L-BFGS-B algorithm to optimize the acquisition function with 20 restarts. Finally, the optimal design configuration can be selected from the Pareto solution.

All numerical simulations and iterative optimization work are conducted on an Intel Xeon Platinum 8375C@2.9 GHz CPU. Python 3.13 code is used to accomplish the optimization loop program and automate the invoking of 3D modeling, meshing, and simulation software, achieving an unattended optimization framework.

### 4.2. Comparison and Analysis of Optimization Results

**Figure 8:**
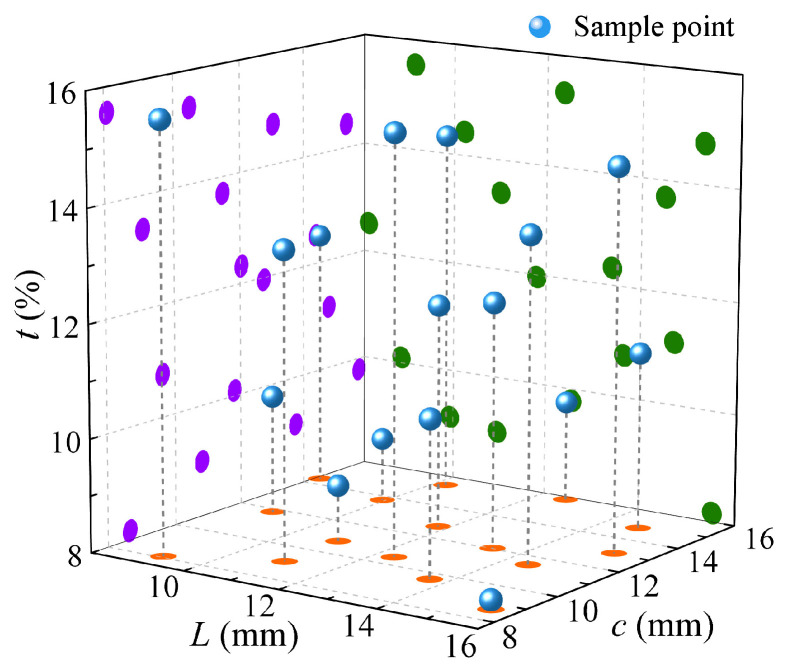
Distribution of samples in geometric design space. (The colored dots mean projections).

This research emphasizes a dual-objective optimization problem, aiming to find better aerodynamic and electromagnetic characteristics. To better explore the distribution of the objective functions in the design space, the Sobol method is used to sample 16 initial samples from the design space using a low-discrepancy sequence, obtaining more uniform sampling points. Figure 8 shows the distribution of the sample points in the geometric design space, indicating that the sample points are relatively uniformly distributed. The uniformity of each dimension is calculated as follows, with the results shown in Table 3: interval standard deviation SD<0.1, uniformity difference $D^{*}<0.1$, and chi-squared homogeneity p>0.05, indicating that the sample points are uniformly distributed. The design parameters of the 16 initial sample points will be used to initialize the sample space and objective function space of Bayesian optimization.

**Figure 9:**
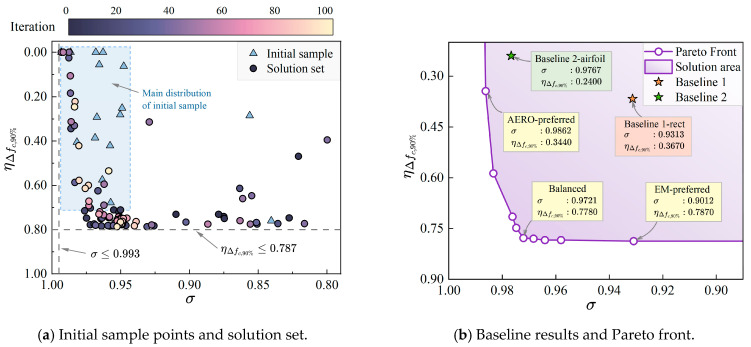
Baseline and Bayesian optimization solution sets.

After generating the initial 16 sample points and calculating their corresponding objective functions, the distribution of the initial sample points in the two objective functions is obtained, as shown in Figure 9a by the triangular points. The x and y axes of the figure represent the aerodynamic and absorption performance of the RIG, respectively. It can be seen that the solution set of the initial sample points in the uniform design space is not uniform but mainly distributed in the region with high aerodynamic characteristics, as shown by the blue region in the figure. Only one sample point is located in the region with high absorption performance.

Calculating the objective functions of the initial sample points, the Bayesian optimization process described earlier is conducted. By predicting the parameters that maximize the expected hypervolume, the next sampling point is obtained, and its objective function is calculated, and so on. The objective function solution sets of 100 optimization sampling points are sequentially calculated, and Figure 9a shows the scatter plot of the solution sets after 100 iterations. It can be seen that the solution set region is within the range of σ∈[0.800,0.993] and $\eta_{\Delta f_{c,90\%}}\in [0,0.787]$. The distribution characteristics of the optimization solution set mainly show an L-shaped distribution. In terms of the iteration sequence, the optimization process first balances exploration and exploitation, and the optimization solution sets mostly reach the optimal values before the 80th generation. The solutions from the 80th to 100th generations tend to explore unknown regions, resulting in some suboptimal solutions and stagnation.

**Figure 10:**
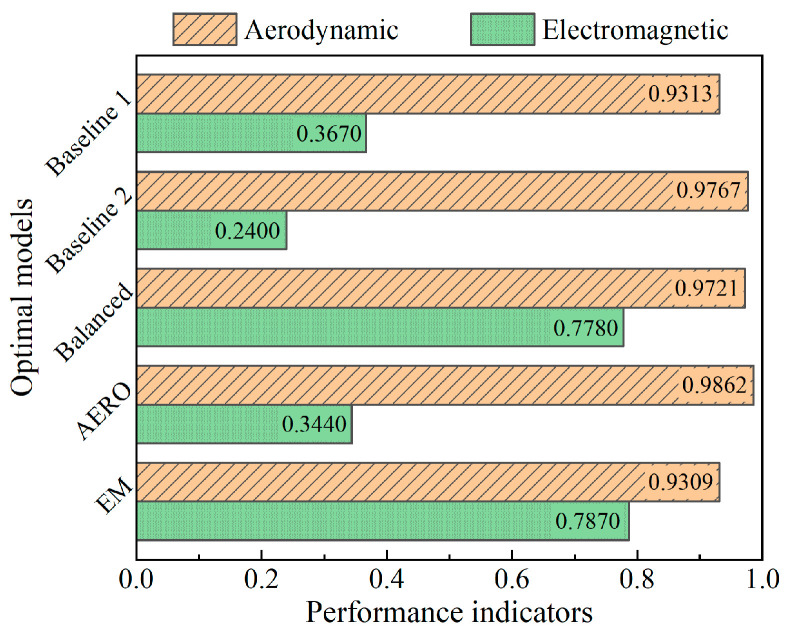
Objective functions of baseline and optimized models.

Since aerodynamic and electromagnetic performance are often conflicting optimization objectives in aerospace applications, it is unlikely that a set of design parameters exists that simultaneously satisfies the optimal values of both objective functions. The non-dominated solutions are selected from Figure 9a and plotted as the Pareto front in Figure 9b, with the feasible region shown in purple. To further explore the optimization results, three optimal results are selected from the Pareto front solution set: aerodynamic-preferred, electromagnetic-preferred, and balanced. Additionally, two baseline results are selected for comparison: Baseline 1 with a rectangular cross-section grille and Baseline 2 with an NACA airfoil cross-section grille, both with the same maximum thickness. The design parameters and objective functions of the typical optimization results and baselines are listed in Table 4 and Figure 10.

A comparison of the design parameters between the rectangular RIG (Baseline 1) and the airfoil-shaped RIG (Baseline 2) reveals that they have identical lengths, depths, maximum thicknesses, and sheet resistance, differing only in their cross-sectional profiles. However, their performance exhibits significant disparities: the total pressure recovery coefficient of the airfoil-shaped RIG increases from 0.9313 to 0.9767, while the absorption bandwidth ratio decreases from 0.3670 to 0.2400. This indicates that, compared to the conventional rectangular RIG with the same design dimensions, the streamlined airfoil profile substantially enhances the airflow work capacity, but the curved metal strip array on the RIG surface reduces the absorption performance.

**Figure 11:**
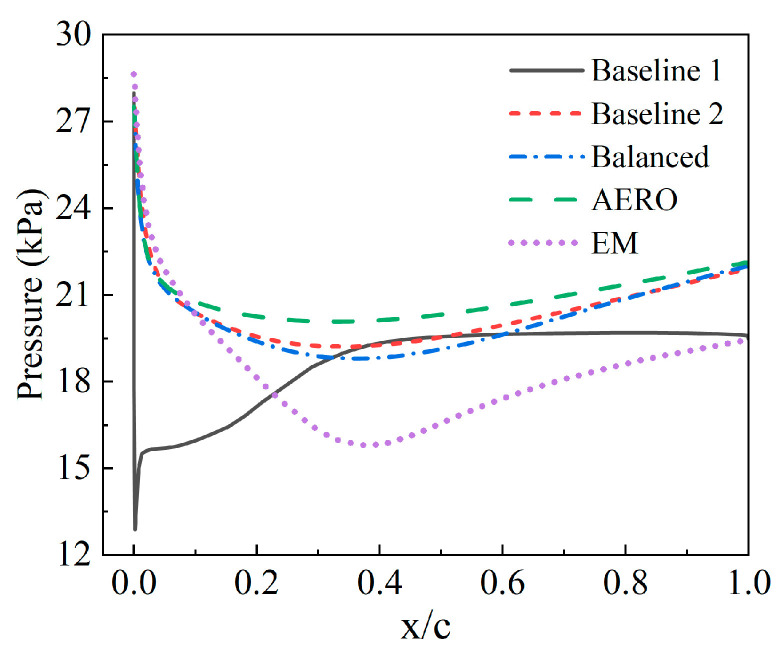
Pressure distribution curves along the airfoil surface for different models.

To further compare the baseline and optimized results, the pressure distribution curves along the symmetric airfoil cross-sections of different models are extracted, as shown in Figure 11. For Baseline 1, the rectangular airfoil’s sharp right-angle edges lead to a sharp pressure drop along the non-dimensional chord length, suggesting potential stall phenomena near the leading edge. This is followed by a pronounced and abrupt pressure rise, accompanied by localized flow separation near the leading edge. Beyond a non-dimensional chord length of 0.4, the surface pressure exhibits a relatively steady decline but lacks a reasonable pressure recovery process. In contrast, Baseline 2 demonstrates a smoother pressure distribution. The pressure curve near the leading edge declines gently and transitions to a smooth recovery beyond a non-dimensional chord length of 0.35. The entire process is more aerodynamically streamlined, resulting in lower flow losses, which aligns with the total pressure recovery coefficient trends in Table 4.

The three optimized models exhibit pressure distribution patterns similar to Baseline 2, with the pressure smoothly decreasing along the non-dimensional chord length and recovering beyond 0.35. However, the balanced model shows a larger pressure drop near the leading edge and a correspondingly greater mid-chord recovery compared to Baseline 2, while the trailing-edge pressure distribution remains consistent. Consequently, the total pressure recovery coefficient of the balanced model closely matches that of Baseline 2. The AERO model demonstrates superior pressure distribution compared to the balanced and Baseline 2 models, whereas the EM model underperforms relative to the other airfoil configurations.

**Figure 12:**
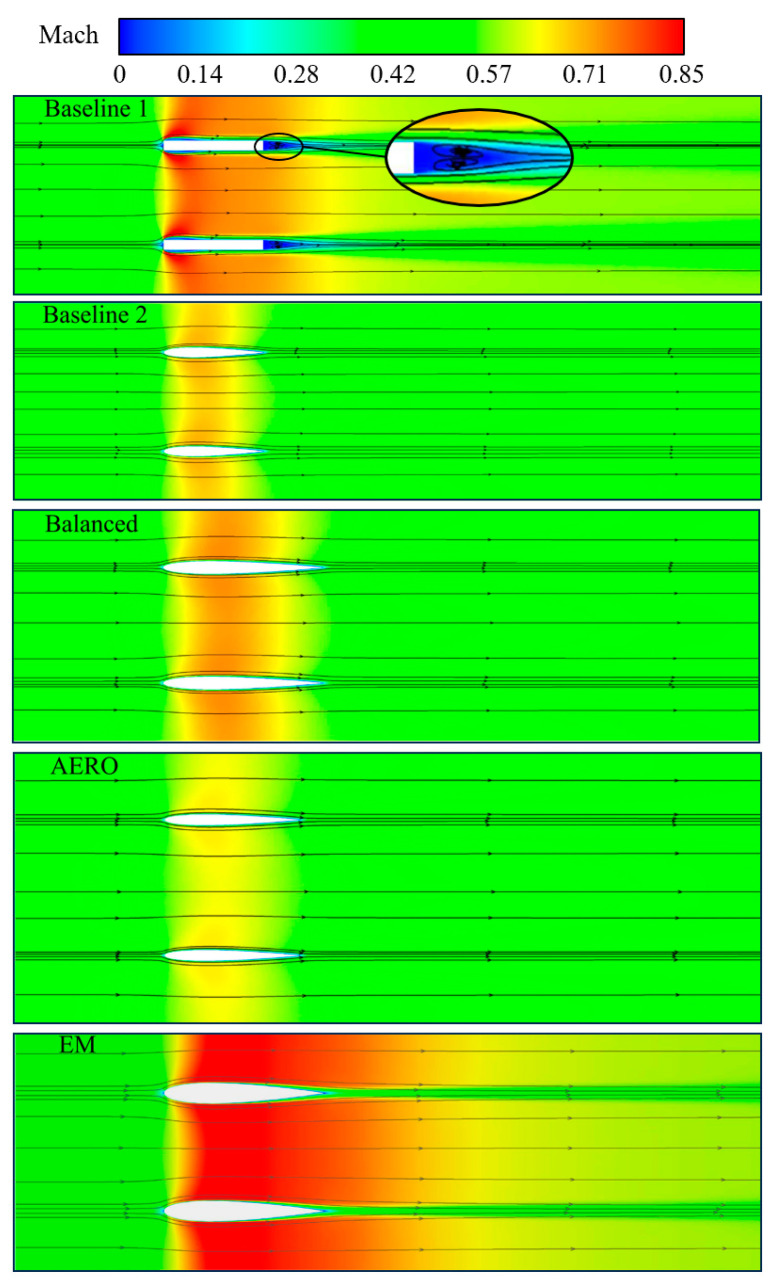
Mach number contours and streamline distribution.

By analyzing the Mach number contours and streamline diagrams of the flow cross-sections of the different models in Figure 12, it can be observed that, when the airflow passes through Baseline 1, a significant velocity stagnation zone is formed due to the flat leading edge of the rectangular airfoil. At the same time, there is strong geometric discontinuity near the leading edge of the airfoil, causing significant airflow acceleration and separation on the upper and lower surfaces near the leading edge, which greatly reduces the work capacity of the airflow passing through the RIG. Additionally, the deflection and separation of the airflow create aerodynamic throats on the upper and lower surfaces near the leading edge, leading to an increase in the Mach number and some aerodynamic losses. Due to the same geometric discontinuity, more pronounced flow separation occurs near the trailing edge, as shown in the local magnification diagram, where two counter-rotating vortex structures are generated, further increasing the total pressure loss of the airflow.

Compared to the rectangular airfoil-shaped RIG Baseline 1, the NACA airfoil Baseline 2 has the same airfoil spacing, thickness, and chord length, but the Mach number distributions of the two are significantly different. The smooth leading edge shape of the NACA airfoil results in a smaller increase in the Mach number than in the former, and the maximum Mach number is located near the maximum airfoil thickness, i.e., near the geometric throat. The Mach number gradually recovers after passing the maximum airfoil thickness and returns to a lower level near the trailing edge. Compared to Baseline 1, Baseline 2 exhibits significantly superior aerodynamic performance.

For the three optimization results, it can be seen that the balanced model has a larger airfoil chord height and chord length than Baseline 2 but does not produce flow separation, resulting in slightly higher viscous losses. The AERO model has the largest airfoil spacing among the models and a relatively low chord height, resulting in lower flow losses. The EM model has the largest relative chord height and chord length and small airfoil spacing, leading to significant airflow acceleration and a slow Mach number recovery process, which results in larger pressure gradients and viscous losses. The analysis of these processes is consistent with the pressure distribution along the airfoil surface shown in Figure 11.

**Figure 13:**
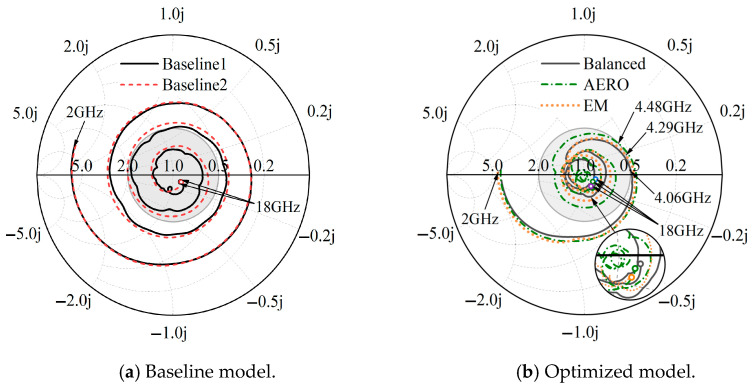
Smith chart of baseline and optimized models.

From the Smith chart shown in Figure 13, the 2 GHz frequency starting points of Baseline 1 and Baseline 2 both occur near the real axis in the inductive region and rotate counterclockwise, passing through the inductive and capacitive regions, and they finally reach the capacitive region at the 18 GHz frequency endpoint. The gray circle in the figure is a constant SWR circle with (1 + j0) as the center and passing through the point (2 + j0). Generally, SWR < 2 is considered a good match. From Figure 13a, it can be seen that the impedance matching characteristics of Baseline 1 and Baseline 2 are similar, indicating that their electrical and physical lengths are similar, which corresponds to the design parameters of the baselines. At the same time, the impedance trajectory of Baseline 2 is always slightly farther from the center than that of Baseline 1, with fewer frequency points in the constant SWR circle, indicating that the impedance matching characteristics of Baseline 2 are inferior to those of Baseline 1. This may be due to the non-uniform thickness of the metal strip array along the electromagnetic wave propagation direction.

As can be observed in Figure 13b, the impedance matching patterns of the three optimized designs are similar to those of the baseline results, starting from the inductive region, passing through the inductive and capacitive regions, and finally reaching the capacitive region at the 18 GHz frequency endpoint. However, the impedance matching effects of the three optimized designs are significantly improved compared to the baseline designs, with more frequency points concentrated within the constant SWR circle. The intersection points of the balanced, AERO, and EM designs with the constant SWR circle are 4.06 GHz, 4.48 GHz, and 4.29 GHz, respectively, compared to 10.21 GHz (Baseline 1) and 11.28 GHz (Baseline 2). This indicates that the optimized results correspond to better impedance matching characteristics. The impedance curve of the balanced design starts from 2 GHz and is located inside the other curves until higher frequencies, where the AERO design’s impedance curve is closer to the center. Therefore, compared to the other optimized designs, the balanced design has better impedance matching characteristics at medium and low frequencies.

**Figure 14:**
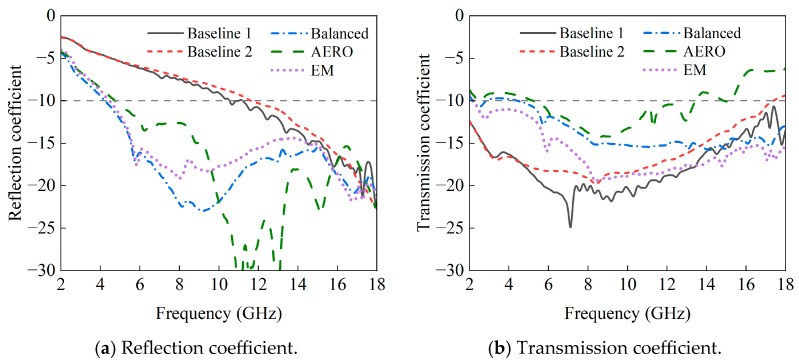
Reflection and transmission coefficient curves of different models.

The reflection and transmission coefficients of the baseline and optimized models are shown in Figure 14. From the reflection coefficients, the reflection coefficient of Baseline 2 is similar to that of Baseline 1 at low frequencies but is slightly higher than that of Baseline 1 in most frequency bands as the frequency increases. For the three optimized results, from 2 GHz to 16 GHz, the reflection coefficients of the balanced, AERO, and EM designs are always lower than those of any baseline results, only exceeding the baselines in the oscillation region from 16 to 18 GHz. Although the AERO design forms a low-reflection interval due to impedance matching in the 10–13 GHz range, it performs poorly in the 6–10 GHz and 16–18 GHz ranges. The reflection coefficients of the balanced and EM designs almost coincide in the 2–6 GHz range, with the balanced design having lower reflection coefficients in the 6–16 GHz range and the EM design performing better in the 16–18 GHz range.

From the figure of the transmission coefficient, the transmission coefficient of Baseline 1 is below −10 dB in the entire frequency range, while that of Baseline 2 is only slightly above −10 dB after 17 GHz. The transmission coefficient of Baseline 2 is always higher than that of Baseline 1 starting from 5 GHz, and, since the reflection coefficient is also higher than that of Baseline 1, this means that the absorption rate will be lower than that of Baseline 1. In the optimized results, all models have transmission coefficients above −10 dB near 2 GHz. The AERO model’s transmission coefficient exceeds −10 dB again starting from 13.4 GHz, meaning that its absorption rate in this frequency band will be less than 0.9. The transmission rate of the EM design is always lower than that of the balanced design after 2.5 GHz, resulting in relatively better absorption performance.

**Figure 15:**
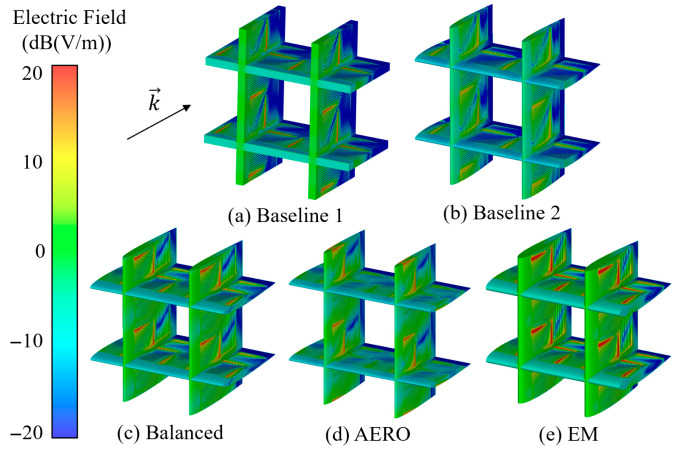
Electric field distribution contours (@ 10 GHz).

The electric field distribution contour of the RIG model at 10 GHz is shown in Figure 15. For ease of observation, the electric field contours of the RIGs have been scaled to the same size. Comparing Baseline 1 and Baseline 2, it can be seen that Baseline 1 has a stronger electric field intensity, indicating that the metal strip array on the Baseline 1 model can excite relatively stronger local plasmon regions, resulting in stronger electromagnetic wave confinement. However, there are large areas of low electric field intensity near the trailing edges of Baseline 1 and Baseline 2, indicating that strong electric fields are not excited in this range. Compared with Figure 13, this phenomenon is due to the poor impedance matching effect, which enhances electromagnetic wave reflection, preventing a large number of electromagnetic waves from entering the RIG.

Therefore, the small number of incident electromagnetic waves elicits a low electric field intensity near the trailing edge. Comparing the optimized results, it can be seen that the AERO model has lower electric field intensity on the metal strip array, indicating weaker plasmon excitation. The balanced and EM models have strong electromagnetic field intensities in local areas of the metal strip array, indicating significant plasmon resonance phenomena in these areas. This confines the energy of the electromagnetic waves near the interface, further enhancing electromagnetic wave dissipation. At the same time, it can be seen that the regions of low electric field intensity in the optimized models are reduced compared to the baseline models, indicating that the impedance matching characteristics of the optimized models are improved, which is consistent with the reflection coefficient curves in Figure 14.

**Figure 16:**
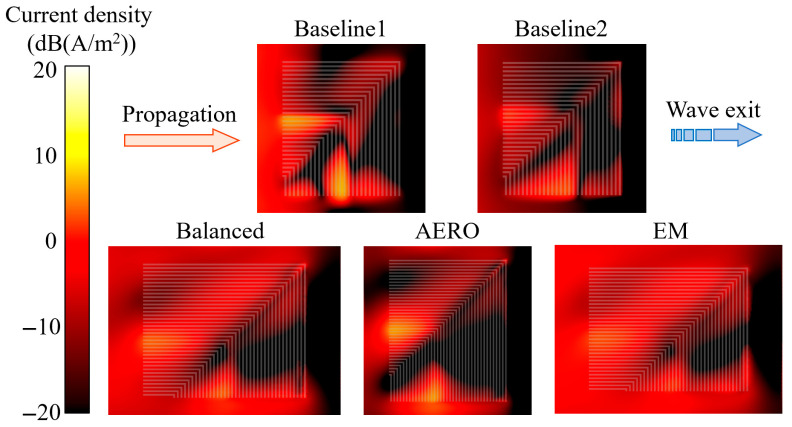
Current density distribution contours (@ 10 GHz).

The current density contour of the ITO film at 10 GHz is depicted in Figure 16. For ease of comparison, the ITO film dimensions in the figure have been scaled to the same height. The resistive film dimensions and sheet resistance of Baseline 1 and Baseline 2 are identical, but the induced current densities are significantly different. Baseline 1 has a higher induced current density, while Baseline 2 has a lower induced current density. The regions with a high induced current density among the two are also different, with the former concentrated between several metal strips and near the leading edge, while the latter is distributed in the short metal strip regions, showing a distinct triangular pattern. Comparing the geometric models of the two, the main difference is that the metal strip array of Baseline 1 is on the same plane, with consistent spacing from the resistive film, while the metal strip array of Baseline 2 is distributed along the curved surface of the airfoil, with varying spacing from the resistive film, resulting in different current density distribution characteristics. The optimized models are similar to Baseline 2, with regions of high induced current density showing a triangular distribution pattern, but the upper triangular regions of the optimized models have a larger current density distribution range, resulting in greater electromagnetic wave dissipation. Additionally, the sheet resistance of the optimized models is higher than 100 Ω/□ of the baseline models, so the resistive films of the optimized models have stronger electromagnetic wave dissipation capabilities under the same current density.

**Figure 17:**
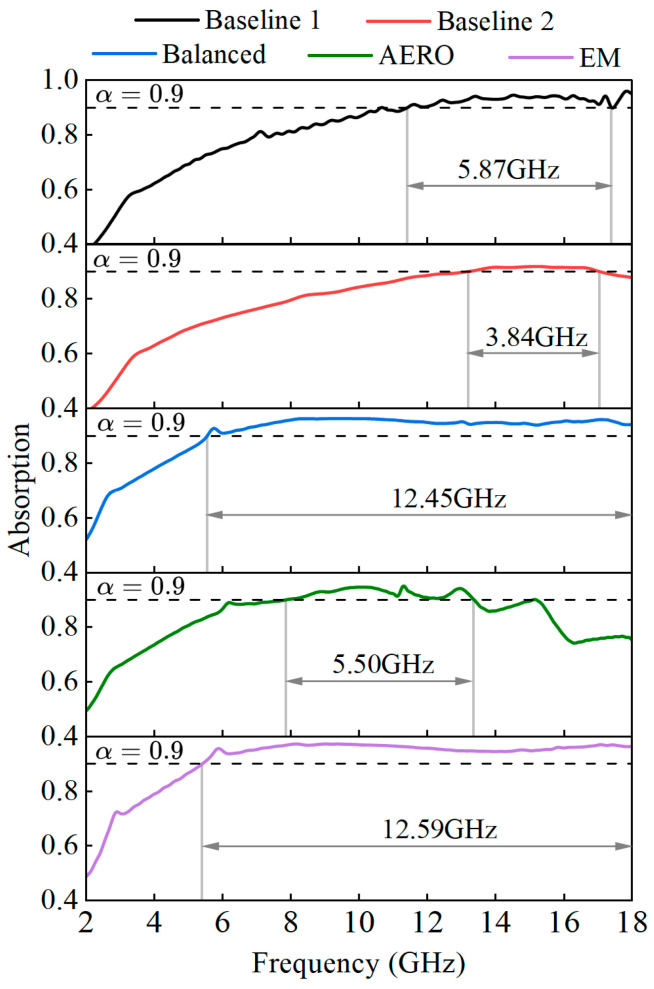
Absorption rates of baseline and optimized models.

The absorption rates of the models in the 2–18 GHz operating frequency band are shown in Figure 17. From the absorption rate variation with frequency for different models, it can be seen that the absorption rate generally shows a trend of first increasing and then decreasing. From the reflection and transmission coefficient curves, it can be seen that the reflection coefficient generally shows a decreasing trend; this phenomenon is mainly due to the recovery of the transmission coefficient after 8–12 GHz. The horizontal dashed line in the figure represents an absorption rate of α=0.9, and the maximum absorption bandwidth with an absorption rate greater than 0.9 is given in each figure. The absorption bandwidths of Baseline 1 and Baseline 2 are 5.87 GHz and 3.84 GHz, respectively, with the absorption range mainly concentrated in the high-frequency band. The range of Baseline 2 is smaller than that of Baseline 1, and the absorption rates of both drop below 0.9 near 18 GHz. The optimized models show better absorption characteristics. The AERO model has the lowest absorption bandwidth among the optimized models at 5.50 GHz due to a significant increase in the transmission coefficient in the high-frequency band. However, this value is still higher than that of Baseline 2 and close to that of Baseline 1. The EM and balanced models have higher absorption bandwidths of 12.59 GHz and 12.45 GHz, respectively, significantly higher than all baseline models. The distribution patterns of the two are similar, with the absorption rate increasing to above α=0.9 near 6 GHz and smoothly varying within the range of α∈[0.9,1.0]. Compared to Baseline 1, the absorption bandwidths increase by 114.4% and 112.0%, respectively. Compared to Baseline 2, the absorption bandwidths increase by 227.9% and 224.2%, respectively.

### 4.3. Analysis of Inlet Loaded with Balanced Model

**Figure 18:**
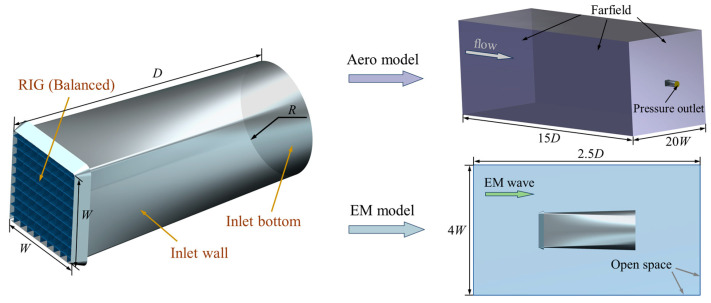
Air intake loaded with balanced model and calculation region.

To study the aerodynamic and electromagnetic characteristics of the balanced model loaded on the inlet, the model is arrayed and installed in a square-to-circular inlet, with the geometric configuration and dimensions of the inlet shown in Figure 18. Finally, the balanced periodic model is arrayed, forming a full model, which is loaded into the inlet to become an integrated coupling inlet–grille model. The specific parameter definitions are shown in Table 5.

**Figure 19:**
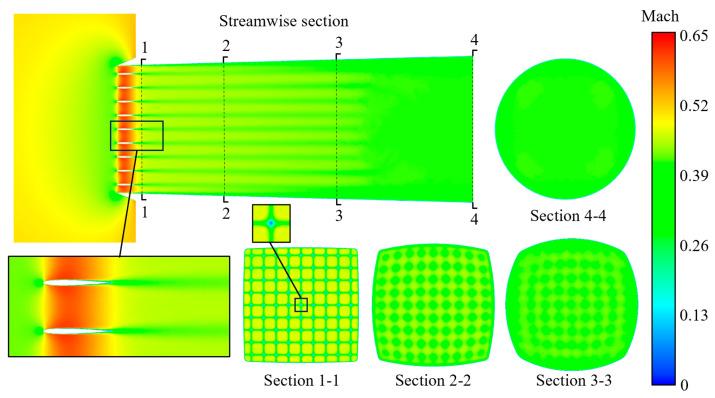
Mach number distribution contours along the cross-sections of the integrated model.

The computational domain and boundary condition settings for the aerodynamic characteristic simulation of the integrated coupling model are shown in Figure 18 and Table 5. The Mach number distribution contour of the inlet with the RIG under these boundary conditions is illustrated in Figure 18. A flow deceleration zone is formed near the inlet of the streamwise section, followed by a flow acceleration zone as the airflow passes through the RIG, and a low-Mach-number wake is formed after passing through the RIG. Comparing the local magnification of the streamwise section with Figure 19, it can be seen that the periodic flow model of the RIG is similar in terms of flow characteristics to the model of the complete inlet, with airflow acceleration formed in the vane channel contraction zone. Sections 1-1, 2-2, 3-3, and 4-4 are cross-sections at 20 mm, 100 mm, 200 mm from the inlet and the outlet, respectively. From each section, it can be seen that the deep blue dotted low-Mach-number wake zone at Section 1-1 rapidly diffuses into the large green medium–low-Mach-number zone at Section 2-2 and then gradually diffuses uniformly at Sections 3-3 and 4-4. Although the outlet conditions and geometric shapes are slightly different from the flow-through model, the overall flow characteristics remain consistent. The total pressure recovery coefficient of the integrated coupling model is 0.9826, slightly higher than the value of 0.9721 for the flow-through periodic balanced model, where the integrated coupling model and the balanced model use different boundary conditions. The total pressure recovery coefficient of the clear model is 0.9930.

**Figure 20:**
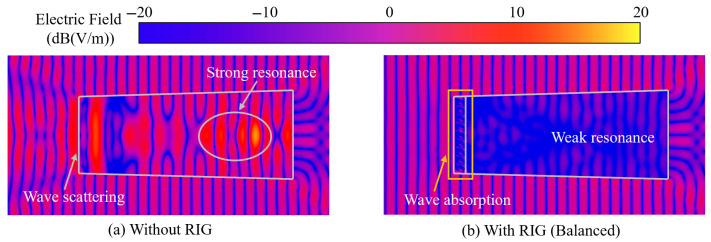
Electric field distribution contours of the cross-sections of the integrated model (0°).

From Figure 20, it can be seen that, when a 10 GHz plane wave is incident on a conventional clean inlet, the electric field intensity is significantly enhanced at multiple positions in the cavity, indicating that strong resonance phenomena occur in the cavity. At the inlet, it can be observed that the superimposed electric field is already affected by the reflected electromagnetic waves. The geometric structure of the inlet causes wave scattering, changing the propagation direction of the plane wave from the planar propagation of the incident wave to the horn-shaped propagation of the reflected wave.

When the plane wave is incident on the integrated coupling model, electromagnetic wave absorption occurs at the RIG. Weak resonance phenomena can be observed in the cavity, with a relatively uniform electric field intensity distribution and a significant reduction in regions of high electric field intensity. The superimposed field at the inlet is almost unchanged, indicating that the reflected echo signal intensity is low. This analysis shows that the electromagnetic waves are largely absorbed at the RIG, and the arrayed balanced model has good absorption performance.

**Figure 21:**
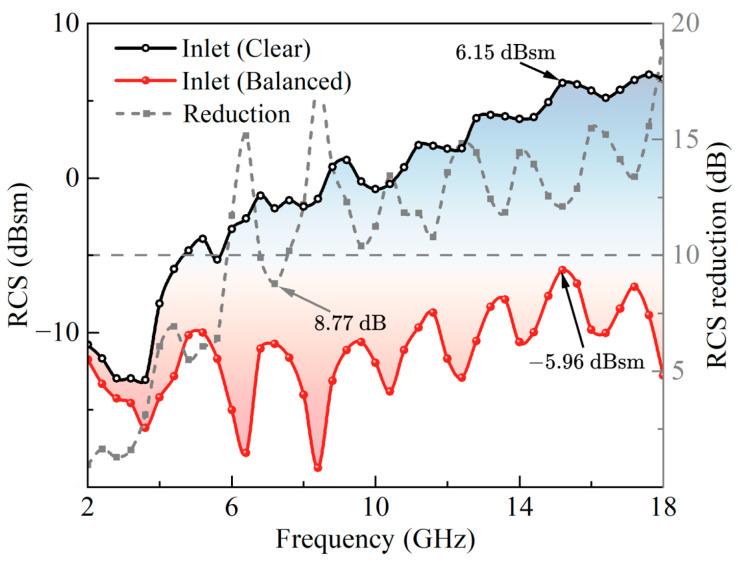
RCS and its reduction values in different models.

The radar cross-section calculations were performed at normal incidence (0° detection angle), where horizontal and vertical polarizations yielded similar results due to the symmetry of the electromagnetic interaction. For this reason, polarization specification was not differentiated in our analysis. The RCS distribution curves of the inlet model under plane wave incidence from 2 to 18 GHz are shown in Figure 21. The RCS of the clear inlet shows a significant oscillatory upward trend as the frequency increases, while the RCS of the inlet with the RIG shows a wide oscillatory variation pattern as the frequency increases. From the RCS reduction values, it can be seen that, from 7.6 GHz to 18 GHz, the RCS reduction of the inlet with the RIG exceeds 10 dB, with a significant decrease in the RCS. In the 2–18 GHz frequency band, the maximum RCS of the clear inlet is 6.15 dBsm@15.2 GHz, while the maximum RCS of the inlet with the RIG is −5.96 dBsm@15.2 GHz. The absorption rate of the balanced model is above 0.9 after about 6 GHz. The RCS reduction of the coupling model is above 10 dB after 6 GHz, but a reduction of 8.77 dB occurs near 7.6 GHz, indicating that the coupling model may weaken the absorption performance of the RIG due to diffraction at the inlet lip edge. However, overall, the RCS reduction of the coupling model is consistent with the absorption rate variation of the corresponding unit model. This indicates that the RIG studied in this paper still has good absorption performance when loaded on the inlet, with the ability to reduce the inlet RCS.

**Figure 22:**
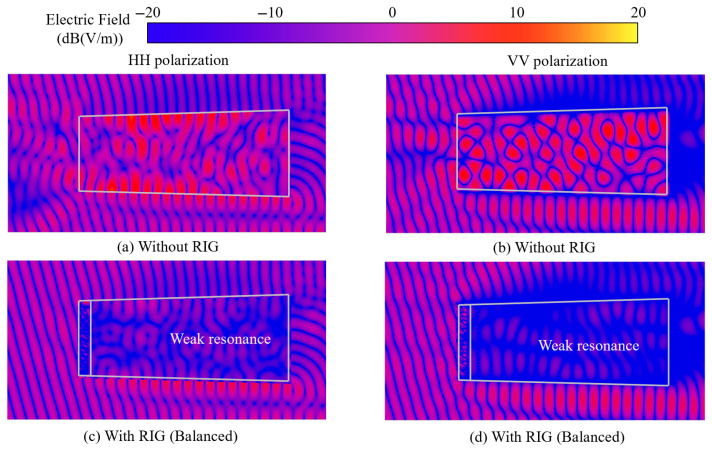
Electric field distribution contours of the cross-sections of the integrated model (15°).

As is shown in Figure 22, when plane waves are incident on the clear model at 15° oblique incidence, intense resonance phenomena occur within the cavity, forming distinct field pattern configurations dependent on the polarization modes. However, the composite field strength remains lower than in the specular reflection case observed at 0° normal incidence. When the plane wave impinges on the integrated coupling model at a 15° oblique angle, the RIG similarly exhibits significant electromagnetic wave absorption. Only weak-resonance regions can be observed within the cavity, with HH polarization demonstrating a slightly higher electric field intensity than VV polarization, although both polarization conditions maintain relatively uniform field distributions with substantially reduced high-intensity regions. The superimposed field at the inlet maintains a comparatively low magnitude, indicating minimal reflected echo signal strength. This suggests that electromagnetic waves neither substantially enter the cavity nor generate significant backscattered reflection.

**Figure 23:**
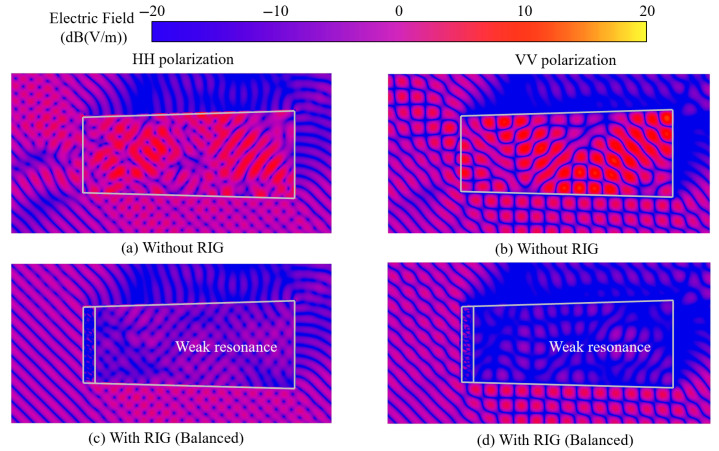
Electric field distribution contours of the cross-sections of the integrated model (45°).

As is depicted in Figure 23, when plane waves are incident on the clear model at 45° oblique incidence, intense resonance occurs within the cavity, with HH and VV polarizations generating two distinct field pattern configurations on the cross-section profile. Distinct reflection and phase interference phenomena of the plane wave can be observed within the cavity. When the plane wave impinges on the integrated coupling model at a 45° oblique angle, the RIG demonstrates significant electromagnetic absorption capabilities. Weak-resonance regions remain observable within the cavity; however, the electric field intensity under HH polarization exceeds that of VV polarization, suggesting that a limited portion of electromagnetic energy transmits through and undergoes multiple scattering events within the cavity. The superimposed field at the inlet maintains a relatively low magnitude, indicating minimal reflection and a low echo signal intensity. The electric field distribution contours under oblique incidence conditions demonstrate that the RIG maintains appreciable absorption performance across various oblique incidence angles.

**Figure 24:**
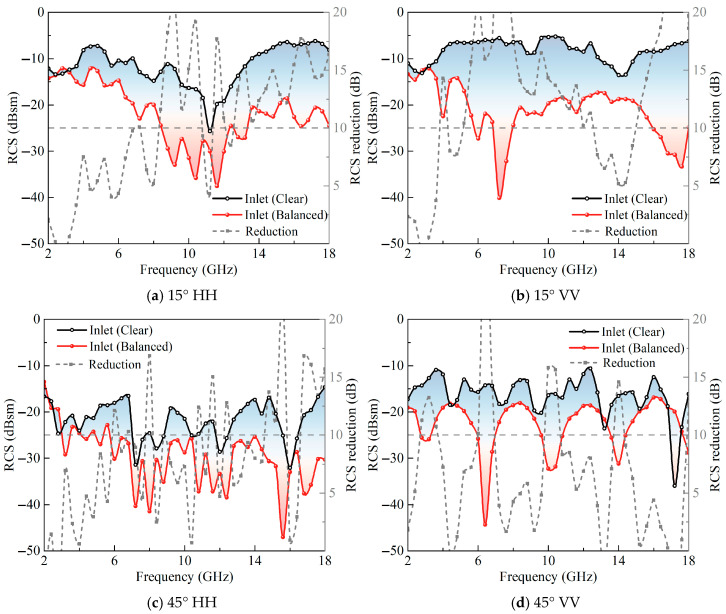
Electric field distribution contours of the cross-sections of the integrated model.

Figure 24 illustrates the RCS distribution curves of the inlet model under plane wave incidence at 15° and 45° oblique angles for both HH and VV polarizations. At the 15° oblique incidence angle, the RCS of the clear model in both HH and VV polarizations remains below −5 dBsm across the 2–18 GHz frequency range, although their frequency-dependent trends exhibit slight variations. The former displays an initial decrease followed by a subsequent increase, while the latter oscillates within a certain range. The balanced model under HH polarization demonstrates a trend similar to the clear model—initially decreasing and subsequently increasing—whereas, under VV polarization, the balanced model exhibits a decrease–increase–decrease pattern. At 15° oblique incidence, the RCS values in the low-frequency band (2–4 GHz) for both polarizations closely approximate those of the clear model, occasionally presenting slightly higher values. This primarily results from the RIG’s limited absorption efficiency in the low-frequency range, leading to stronger reflections, with electromagnetic waves not experiencing the oscillation and scattering effects within the cavity, thus minimally influencing the RCS. Due to the vectorial superposition effect of the electromagnetic phase on the RCS under oblique incidence, the RCS reduction values exhibit pronounced oscillatory characteristics; nevertheless, it generally maintains values exceeding 4 dBsm across the 4–18 GHz frequency range.

As the oblique incidence angle further increases to 45°, the RCS of the clear model for both HH and VV polarizations decreases further, remaining below −10 dBsm throughout the 2–18 GHz frequency range. With increasing frequencies, the RCS values for both HH and VV polarizations oscillate significantly, without exhibiting distinct patterns. In the low-frequency range (2–4 GHz), the RCS of the coupled model under HH polarization approximates that of the clear model, showing no significant reduction and occasionally exhibiting slight increases.

Within the 4–18 GHz frequency range, the RCS of the coupled model under HH polarization consistently maintains low values, predominantly below −20 dBsm, while the relative RCS reduction deteriorates, maintaining values only slightly above 0 dBsm. The RCS of the coupled model under VV polarization displays undulating variations, with significant reductions relative to the clear model across most frequencies, although the low RCS points of the clear model occasionally result in RCS reductions of less than 0 dBsm. This primarily occurs because the clear model’s RCS baseline is already relatively low, with phase-induced localized minima diminishing the RCS reduction values. Despite instances of RCS increases at specific frequency points, the overall RCS curve demonstrates a notable reduction compared to the clear model. Evidently, compared to normal electromagnetic wave incidence, oblique incidence results in degraded RCS reduction at certain frequency points; nevertheless, the coupled model’s RCS curve generally exhibits superior performance compared to the clear model.

## 5. Conclusions

To address the high aerodynamic losses associated with conventional absorbing structures in engine inlet grilles, a design based on symmetric airfoils is proposed. To improve the optimization efficiency of aerodynamic and absorption performance, an automatic optimization framework based on multi-objective Bayesian optimization is established. The proposed design is compared with baseline models in terms of comprehensive performance, including the performance of the optimized model loaded on the inlet. The main conclusions are as follows:

(1) The proposed airfoil-shaped radar-absorbing inlet grille, compared with a rectangular one under the same geometric constraints, significantly improves the aerodynamic performance yet reduces the absorption capabilities, with a 66.08% reduction in aerodynamic losses and a 34.60% reduction in the absorption bandwidth.
(2) A multi-objective Bayesian optimization framework suitable for periodic grille aerodynamic/stealth comprehensive optimization is proposed. In the optimization framework, Sobol low-discrepancy sequence sampling is used as the sampling method, Gaussian regression is used as the probabilistic surrogate model, and the EHVI acquisition function is used as the exploration strategy. Compared with traditional design methods, our framework can reduce the need for manual intervention and approach the Pareto front in fewer iterations.
(3) The geometric design parameters of the airfoil-shaped radar-absorbing grille significantly affect the flow field distribution and the aerodynamic and electromagnetic characteristics of the grille, while the sheet resistance of the ITO film has no effect on the aerodynamic performance. Compared with conventional radar-absorbing grilles, the optimized balanced model reduces aerodynamic losses by 57.79% and increases the absorption bandwidth by 111.99%.
(4) Compared with the clear inlet without loading, the integrated coupling model with the optimized model has still low aerodynamic losses and better absorption performance, with the ability to significantly reduce the inlet RCS. When the incident wave is oblique, the integrated coupling model still maintains good performance in reducing the RCS.

Our work provides a feasible design scheme for aircraft inlet radar stealth. By optimizing the original radar-absorbing inlet grille with airfoil design, a significant improvement in stealth performance can be achieved with acceptable aerodynamic losses. This could provide design ideas for future advanced aircraft with non-metallic, lightweight grilles and low-observable inlet systems.

## Figures and Tables

**Table 1:** Design parameters of RIG.

Design Parameter	Symbol	Value
RIG interval	L	8.00 mm
RIG chord length	c	8.00 mm
RIG maximum thickness ratio	t	6.65 mm
Metal strip width	$l_{w}$	0.125 mm
Metal strip interval	$l_{s}$	0.125 mm
Metal strip thickness	$l_{t}$	0.01 mm
ITO film thickness	$t_{PET}$	1.00 mm
ITO film resistance	ρ	100 Ω/□

**Table 2:** Aerodynamic boundary conditions in optimization.

Boundary Condition	Attribute
Pressure farfield	$p_{0}=22,632\;Pa$ $T_{0}=216.65\;K$ Ma=0.5
Pressure outlet	$p_{0}=22,632\;Pa$
Period	$p_{jump}=0\;Pa$
Wall	No slip

**Table 3:** Uniformity metrics of design parameters of sample points.

Design Parameter	SD	$D^{*}$	p
L	0.04	0.06	0.93
c	0.02	0.06	0.99
t	0.03	0.06	0.93
ρ	0.04	0.10	0.93

**Table 4:** Design parameters of baseline and typical Bayesian optimized models.

Model	*L* (mm)	*c* (mm)	*t* (mm)	ρ (Ω/sq)
Baseline 1	10.00	10.00	10.00	100.00
Baseline 2	10.00	10.00	10.00	100.00
Balanced	11.62	16.00	8.00	375.27
AERO	13.63	13.56	8.00	461.90
EM	11.85	16.00	12.83	292.31

**Table 5:** Geometric design parameters of aircraft intake.

Design Parameter	Symbol	Value (mm)
Width of inlet	W	105
Length of intake	D	300
Radius of intake interface	R	59

## Data Availability

The original contributions presented in this study are included in the article. Further inquiries can be directed to the corresponding author.
